# Complete Genome Sequence of *Xanthomonas campestris* pv. campestris Strain 17 from Taiwan

**DOI:** 10.1128/genomeA.01466-15

**Published:** 2015-12-17

**Authors:** Yi-Chung Liu, Shiuh-Chaun Wang, Yu-Jen Yu, Kit-Man Fung, Ming-Te Yang, Yi-Hsiung Tseng, Shih-Feng Tsai, H. Sunny Sun, Ping-Chiang Lyu, Shan-Ho Chou

**Affiliations:** a Institute of Population Sciences, National Health Research Institutes, Miaoli, Taiwan; b Center for Biomedical Resources, National Health Research Institutes, Miaoli, Taiwan; c Institute of Molecular Biology, National Chung Hsing University, Taichung, Taiwan; d Institute of Molecular and Genomic Medicine, National Health Research Institutes, Miaoli, Taiwan; e Institute of Molecular Medicine, College of Medicine, National Cheng Kung University, Tainan, Taiwan; f Institute of Bioinformatics and Structural Biology, National Tsing Hua University, Hsinchu, Taiwan; g Institute of Biochemistry and NCHU Biotechnological Center, National Chung Hsing University, Taichung, Taiwan

## Abstract

*Xanthomonas campestris* pv. campestris 17 is a Gram-negative bacterium that is phytopathogenic to cruciferous plants in Taiwan. The 4,994,426-bp-long genome consists of 24 contigs with 4,050 protein-coding genes, 1 noncoding RNA (ncRNA) gene, 6 rRNA genes, and 55 tRNA genes.

## GENOME ANNOUNCEMENT

*Xanthomonas campestris* pv. campestris is phytopathogenic to cruciferous plants and results in worldwide agricultural losses. It causes black rot, which is one of the most serious plant diseases in Taiwan (1). The warm and wet local climate is suitable for plant infection by *X. campestris* pv. campestris and the development of disease. The cultivation of black rot-resistant cultivars has long been recognized as an important control strategy. Meanwhile, an investigation of the genome sequence from the local strain can be used to discover its genetic and gene expression profiles to prevent black rot disease.

*X. campestris* pv. campestris 17 was isolated from the leaves of the infected plants in Taiwan. Its genome sequencing was initially carried out using the Sanger sequencing method in 2002. After data assembly, 575 contigs consisting of 5,228,421 bp with approximately 10-fold coverage were obtained. The draft sequences were found to contain either 6,327 or 3,811 genes, as predicted by the Glimmer 2.0 (2) or ORPHEUS (3) program, respectively, using *X. campestris* pv. campestris ATCC 33913 as a reference. To improve the previous sequencing result, the genome of *X. campestris* pv. campestris 17 was resequenced on a GAIIx Illumina platform in 2012. Genome assembly was conducted using the SSAKE software (4) and Velvet assembler (5). However, some gaps were still present, which were further completed by PCR and cloning, followed by Sanger sequencing. A total length of 4,994,426 bp, comprising 24 contigs, with a contig *N*_50_ of 722,084 bp and 506.7-fold coverage, were obtained. The genome was found to be enriched with G+C content (65.1%). The sequence was annotated using the RAST server (6) and the NCBI Prokaryotic Genome Automatic Annotation Pipeline (PGAAP) (http://www.ncbi.nlm.nih.gov/genome/annotation_prok), which predicted 4,215 genes, 4,050 coding sequences (CDSs), 1 noncoding RNA (ncRNA) gene, 6 rRNA genes, and 55 tRNA genes. The genome information presented here will help discover the mechanism of *X. campestris* pv. campestris-plant interactions and allow for further investigation of the *X. campestris* pv. campestris functional and structural genomics.

### Nucleotide sequence accession numbers.

The whole-genome sequence has been deposited in GenBank under the accession number CP011946. The version described in this paper is version CP011946.1.
